# Antithrombotic effects and related mechanisms of *Salvia deserta Schang* root EtOAc extracts

**DOI:** 10.1038/s41598-018-36026-7

**Published:** 2018-12-10

**Authors:** Rena Kasimu, Xinling Wang, Xiaomei Wang, Junping Hu, Xiaoqing Wang, Yuming Mu

**Affiliations:** 1 College of Pharmacy, Xinjiang Medical University, No. 393 Xinyi Road, Urumqi, 830011 China; 2 The First Affiliated Hospital Of Xinjiang Medical University, No. 137 South Liyushan Road, Urumqi, 830054 China

## Abstract

*Salvia deserta Schang* (SDS) belongs to the same family as *Salvia miltiorrhiza bunge*, one of the antithrombotic Chinese herbal medicines. In our study, EtOAc root extracts were analyzed for their effects on adenosine diphosphate (ADP)-induced platelet aggregation in rabbits and FeCl_3_-induced rat common carotid artery thrombosis as well as on rat blood plasma concentrations of thromboxane B2 (TXB_2_), 6-keto-prostaglandin F1 alpha (6-keto-PGF_1α_), antithrombin-III (AT-III), protein C (PC), plasminogen (PLG), plasminogen activator inhibitor (PAI-1), von Willebrand factor (vWF) and tissue-type plasminogen activator (t-PA). EtOAc extracts from SDS roots had significant inhibitory effects on ADP-induced maximum platelet aggregation rate (10.2 ± 2.6 vs control 35.7 ± 5.2; *P* < 0.05), reduced the FeCl_3_-induced rat common carotid artery thrombus weight and thrombus area ratio (*P* < 0.05), significantly decreased plasma TXB_2_, vWF and PAI-1 levels and increased 6-keto-PGF_1α_ and t-PA levels in a dose dependent manner (all *P* < 0.05). Thus, the ratio of TXB_2_/6-keto-PGF_1α_ was significantly decreased (*P* < 0.05), while the ratio of t-PA/*P*AI-1 was significantly increased (*P* < 0.05). In addition, enhanced AT-III and PC activities indicated coagulation inactivation effects of EtOAc SDS root extracts. EtOAc extraction from SDS showed antithrombotic effects, which are likely due to platelet adhesion and aggregation inhibition as well as anticoagulant activities.

## Introduction

Numerous genetic, acquired and environmental factors can tip the homeostatic balance in favor of coagulation and thus lead to the formation of thrombi, which is a common pathology underlying ischemic heart disease, stroke and venous thromboembolism. It has been reported that ischemic heart disease and stroke collectively are responsible for one in four deaths worldwide^1,2^. Thus, despite the existing available antithrombotic agents, new effective drugs are still urgently required.

*Salvia deserta Schang* (SDS) is a perennial plant belonging to the *Lamiaceae* family and is widely distributed in the Gobi wilderness of Xinjiang province. SDS is a species of the Salvia genus like *Salvia miltiorrhiza bunge*, whose root extracts are an important Chinese herbal medicine called Danshen, which can affect hemostasis by several mechanisms including inhibition of platelet aggregation, interference with extrinsic blood coagulation, antithrombin III-like activity and promotion of fibrinolytic activity. Therefore, it is commonly used in Chinese clinics as antithrombotic therapy^3–6^. However, whether SDS extracts also have anti-thrombotic effects has rarely been investigated.

The chemical composition of whole plant SDS was previously systematically studied. About 30 different compounds including phenolic acids, diterpenoid quinones, flavonoids, triterpenoids and others were isolated of which the hydrosoluble phenolic acids and liposoluble terpenoids were also found in *Salvia miltiorrhiza bunge*^7–9^. The diterpenoid quinones 6,7-dehydroxyleanone and 6,7-dehydroroyleanone have effects in preventing myocardial ischemia, inhibiting platelet aggregation and inducing nitric oxide synthase *in vitro*^10,11^, and the triterpenoid oleanolic acid can significantly inhibit collagen and ADP-induced platelet aggregation to protect the heart^12,13^. In the present study, we hypothesized that SDS might have similar antithrombotic effects to *Salvia miltiorrhiza bunge* and we compared the SDS extract and Danshen application outcomes on thrombosis and related factor patterns in rabbit and mouse models.

## Methods and Materials

### Experimental animal ethics

The study was approved by the ethical committee of Xinjiang Medical University and all procedures involving animals were performed in accordance with the ethical standards of the Guidelines for the Humane Treatment of Laboratory Animals (Ministry of Science and Technology of the People’s Republic of China, Policy No. 2006 398).

#### Rabbits

Two hundred SPF grade healthy New Zealand white rabbits were provided by the animal research center of the Xinjiang Medical University (certificate number: SCXK (Xin) 2011-0004). The animals were male and female, weighing 2.0 ± 0.2 kg, and were kept at 21 ± 2 °C, with a light cycle of 12 hours/day at 40–45% humidity and free access to water and food.

#### Rats

One hundred and thirty healthy male Sprague-Dawley (SD) rats (SPF grade), weighing 250–300 g were provided by the experimental animal center of Xinjiang Medical University (license number: SCXK (Xin) 2011-0004) and kept under the same conditions as the rabbits but in separate holding rooms.

### Preparation of different SDS components for different solvent extractions

SDS plants were separated into roots, stems, leaves and flowers, and then dried in the shade and finally pulverized.

### H_2_O extraction

Roots, stems, leaves and flowers were respectively extracted 3 times for 1 hour by a reflux extraction method in water at 80 °C. The extracts were combined, concentrated and freeze-dried to obtain the water extracts of roots, stems, leaves and flowers.

### Ethanol extraction

Roots, stems, leaves and flowers were extracted 3 times using a method involving 95% ethanol, 1 hour per extract; the 3 extracts were combined, concentrated in a low-temperature vacuum under reduced pressure and dried to obtain ethanol extracts of roots, stems, leaves and flowers.

### EtOAc soluble fraction (ESF)

Roots, stems, leaves and flowers were reflux extracted 3 times for 1 hour with acetate (EtOAc); the 3 extracts were combined, concentrated under atmospheric pressure and dried to obtain ESFs of roots, stems, leaves and flowers.

### Adenosine diphosphate (ADP)-induced platelet aggregation test

For each SDS plant component (root, stem, leaves and flower) 50 New Zealand white rabbits were randomly divided into aspirin (10 mg/kg), high (40–50 mg/kg), middle (20–25 mg/kg) and low (10–12.5 mg/kg) SDS extract doses as well as control groups; each group was comprised of 10 rabbits. Intragastric administration was carried out 3 times a day for the controls and SDS low/middle/high dose groups, and once a day for the aspirin group for 3 consecutive days. An additional dose was administered 1 hour before the operation.

For the horminone, 7-O-acetylhorminone and 6,7-dehydeoroyleanone experiments, 1,000 µg/mL, 100 µg/mL and 10 µg/mL of each chemical dissolved in 5% methyl alcohol-saline water was administered to 7 rabbits in each dosage group, with one group for each chemical acting as the control (*n* = 7) (5% methyl alcohol-saline water only).

Blood was collected by cardiac puncture and coagulation prevented by 3.8% sodium citrate (the volume ratio of blood with anticoagulant was 9:1), centrifuged at 1,000 rpm at room temperature for 10 min, after which the upper plasma layer was aspirated as platelet-rich plasma (PRP). The remaining sample was centrifuged again at 4,000 rpm at room temperature for 15 min and the upper plasma layer was aspirated as the platelet-poor plasma (PPP) fraction.

The PRP was adjusted to a platelet concentration of 4 ~ 5 × 10^8^/mL with PPP. The ADP-induced platelet maximum aggregation rate (MAR) was determined using Born’s turbidimetric method^14^. ADP was purchased from Chrono-Log Corp. (Havertown, PA, US: lot number 3427) and data are presented as the maximum aggregation inhibition rate (MAIR) according to the following formula: MAIR (%) = platelet aggregation rate of the control group%- platelet aggregation rate of test drug treated group%/platelet aggregation rate of the control group%.

### FeCl_3_-induced rat common carotid artery thrombosis experiment

#### Different root extraction method measurements

**Figure 1:**
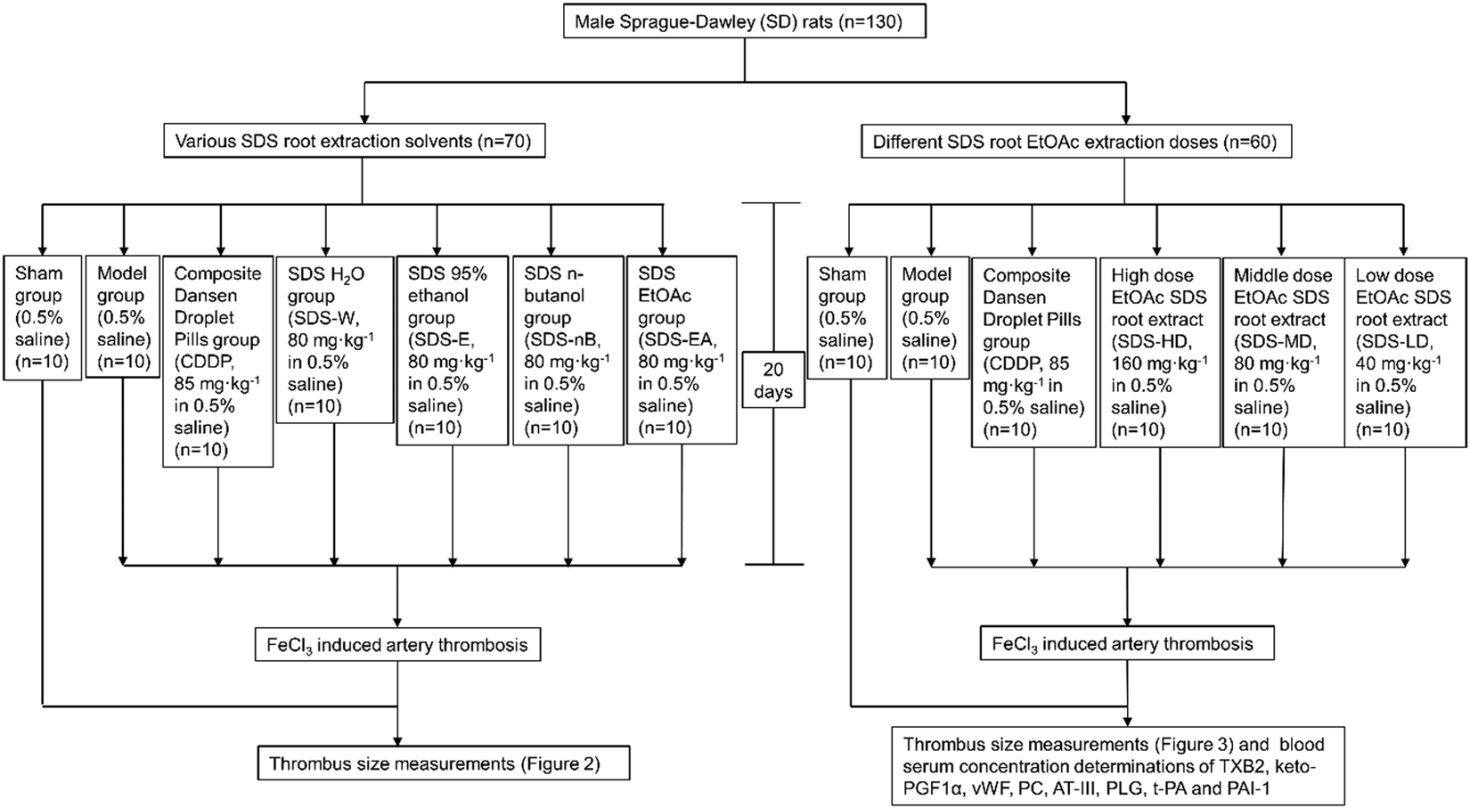
Flow chart of the rat experiments.

Seventy male rats were randomly divided into 7 groups with 10 rats in each group, including a group that did not receive FeCl_3_ (0.5% saline) (Sham), a control group (0.5% saline) (Model), a composite Danshen droplet pills group (Tianjin Tasly Pharmaceutical Group Co., Ltd., batch number: 20130522) dissolved as 85 mg/kg in 0.5% saline as a positive control (CDDP), a SDS water extraction group (SDS-W), 80 mg/kg in 0.5% saline, a SDS 95% ethanol extraction group (SDS-E), 80 mg/kg in 0.5% saline, a SDS n-butanol soluble fraction group (SDS-BuSF), 80 mg/kg in 0.5% saline) and a SDS EtOAc soluble fraction group (SDS-ESF), 80 mg/kg in 0.5% saline. All the animals underwent intragastric administration for 20 days, once daily (Fig. 1).

#### Dose-dependent SDS ESF measurements

For dose-dependent measurements, rats were treated with high (160 mg/kg, SDS-HD), middle (80 mg/kg, SDS-MD) and low (40 mg/kg, SDS-LD) doses of SDS root ESF dissolved in 0.5% saline as well as a no FeCL_3_ group (0.5% saline) (Sham), a control group (0.5% saline) (Model) and a 85 mg/kg composite Danshen droplet pills group dissolved in 0.5% saline as a positive control (CDDP). Again, all the animals underwent intragastric administration for 20 days, once daily (Fig. 1).

### Common carotid artery thrombosis rat model after 20 days of treatment

A common carotid artery thrombosis model was established by reference to previous studies^15–17^. In order to determine the time to blood vessel occlusion (TTO), in preliminary experiments the necessary FeCl_3_ concentration and exposure duration of the common carotid artery have been measured with Doppler ultrasound (Supplementary Table 1, Supplementary Figure 1). Isolated 2 cm segments of the left common carotid artery, removed under deep general anesthesia induced by intraperitoneal injection of urethane (1.5 g/kg), had a small piece of plastic film (3 cm × 1.5 cm) placed under them to protect tissues surrounding the blood vessels. Then a small piece of filter paper (1 cm × 1 cm) soaked in 2.16 mol/L FeCl_3_ solution was applied onto the exposed surface of the artery, which was replaced with saline soaked (filter paper in the sham group. The filter paper was positioned close to the blood vessel wall. The filter paper was removed after 20 min (the start time was when the filter paper was positioned on the artery). The left blood vessel on the site of thrombosis was removed and the residual blood blotted on filter paper and weighted. In addition, the thrombosis arteries were fixed in 10% formalin solution for 24 hours and washed in running water for 4 hours, followed by paraffin sections, HE staining and thrombosis analysis using light microscopy. The thrombus area determination has been performed with image pro plus 6.0 software (Silver Spring, MD, USA)

### Detecting levels of thrombus related factors

Blood was collected via the abdominal aorta of FeCl_3_-induced common carotid artery thrombosis rats and anticoagulated with sodium citrate (1:9) after removal of the thrombosis arteries. The blood was centrifuged at 3,000 rpm at 4 °C for 15 min and the supernatant plasma collected for thrombus factor determinations. TXB_2_ and 6-keto-PGF_1_α radioimmunoassay kits were purchased from North Biotechnology Institute (Beijing, China). PC-C, AT-III, vWF ELISA kits as well as PAI-1 and t-PA test kits, and a PLG immunoassay quantitative test kit were purchased from West Tang Biological Technology Co., Ltd. (Shanghai, China). All measurements were carried out according to the manufacturer’s instructions.

### High performance liquid chromatography (HPLC)/gas chromatography mass spectrometry (CG-MS) SDS extract analysis

Chromatographic separation of SDS root extracts was performed using a Surveyor HPLC system (ThermoFisher Scientific, San Jose, CA, USA) composed of an autosampler and an HPLC pump. The column used was an Atlantis^®^ HILIC Silica, 4.6 mm × 250 mm, 5 µm (Waters Corporation, Milford, MA). The analytes were separated with isocratic elution: mobile phase water (A) methanol solution (B) gradient (0~19 min, 25% A, flow rate 0.8 mL/min; 19~22 min; 25%~13% A, flow rate 0.8~1.0 mL/min; 22~30 min, 13% A, flow rate 1.0 mL/min. Measurement wavelength was 272 nm (0~22.00 min for hominone, 7-O-acetylhorminone), 330 nm (22.01~30.00 min for 6,7-dehydeoroyleanone) and the column temperature was 40 °C with a sample injection volume of 10 μL. For the MS/MS analysis, a TSQ Quantum Ultra triple quadrupole mass spectrometer (ThermoFisher Scientific, San Jose, CA, USA) was used.

### Statistical analysis and data processing

Stataistical analyses were pereformed with SPSS for Windows (Ver. 16.0. Chicago, SPSS Inc.). Continuous variables are presented as mean ± standard deviation ($\bar{X}$ ± SD), Differences between different doses were analysed using one-way ANOVA and comparisons between two groups were performed using bonferroni post hoc-test. The ratio variables were analysed with chi-square test. *P* < 0.05 was considered to be statistically significant.

## Results

### SDS root EtOAc extract significantly inhibited platelet aggregation

First, we investigated whether the different plant parts and extraction methods yielded effective treatments to inhibit platelet aggregation in rabbits (data not shown), when we found that only root ESF inhibited platelet aggregation. Further investigations revealed that MAR/% in high doses of root extracts was 10.2 ± 2.6, compare to MAR/% in control (35.7 ± 5.2), which showed significant inhibition of platelet aggregation (*P* < 0.05) to a similar extent as aspirin (*P* > 0.05) (Table 1).

**Table 1:** Effect of EtOAc extracts of indicated SDS parts on New Zealand white rabbit platelet aggregation ($\bar{X}$ mean ± SD, n = 10).

Faction	Groups	Dose/mg·kg	ADP	
			MAR/%	MAIR/%
Root	**Aspirin**	10	15.2 ± 6.5	57.4 ± 12.9
	Control		35.7 ± 5.2^Δ^	
	STD Low Dose	10	40.8 ± 11.2^Δ^	0.0 ± 0.0^Δ^
	STD Middle Dose	20	29.8 ± 8.7^Δ^	16.5 ± 7.9^Δ^
	STD High Dose	40	10.2 ± 2.6^*^	71.4 ± 4.1^Δ^
	*p-*value (STD dose)		<0.0001	
	*p-*value (Aspirin + STD dose)		<0.0001	<0.0001
Stem	**Aspirin**	10	13.3 ± 8.1	67.2 ± 11.7
	Control		40.6 ± 11.53^Δ^	
	STD Low Dose	10	42.9 ± 17.3^Δ^	0.0 ± 0.0^Δ^
	STD Middle Dose	20	43.1 ± 9.5^Δ^	0.0 ± 0.0^Δ^
	STD High Dose	40	35.5 ± 7.4^Δ^	12.6 ± 5.8^Δ^
	*p-*value (STD dose)		0.4667	
	*p-*value (Aspirin + STD dose)		<0.0001	<0.0001
Leaf	**Aspirin**	10	8.2 ± 3.6	70.8 ± 5.1
	Control		28.1 ± 8.1^Δ^	
	STD Low Dose	10	30.4 ± 10.7^Δ^	0.0 ± 0.0^Δ^
	STD Middle Dose	20	24.5 ± 7.3^Δ^	12.8 ± 1.8^Δ^
	STD High Dose	40	22.8 ± 6.3^Δ^	18.8 ± 2.4^Δ^
	*p-*value (STD dose)		0.1780	
	*p-*value (Aspirin + STD dose)		<0.0001	<0.0001
Flower	**Aspirin**	10	8.2 ± 3.6	70.8 ± 5.1
	Control		28.1 ± 8.1^Δ^	
	STD Low Dose	12.5	35.6 ± 6.6^Δ^	0.0 ± 0.0^Δ^
	STD Middle Dose	25	27.1 ± 7.6^Δ^	3.5 ± 1.6^Δ^
	STD High Dose	50	20.8 ± 2.7^Δ^	25.9 ± 12.3^Δ^
	*p-*value (STD dose)		0.0002	
	*p-*value (Aspirin + STD dose)		<0.0001	<0.0001

*P < 0.05 significant difference compared to control group; ^Δ^*P* < 0.05 significant difference compared to aspirin group.

MAIR(%) = platelet aggregation rate of the control group% - platelet aggregation rate of test drug treated group% / platelet aggregation rate of the control group%.

### Effects of H_2_O, ethanol, n-butanol and EtOAc SDS root extracts on rat carotid artery thrombosis

**Figure 2:**
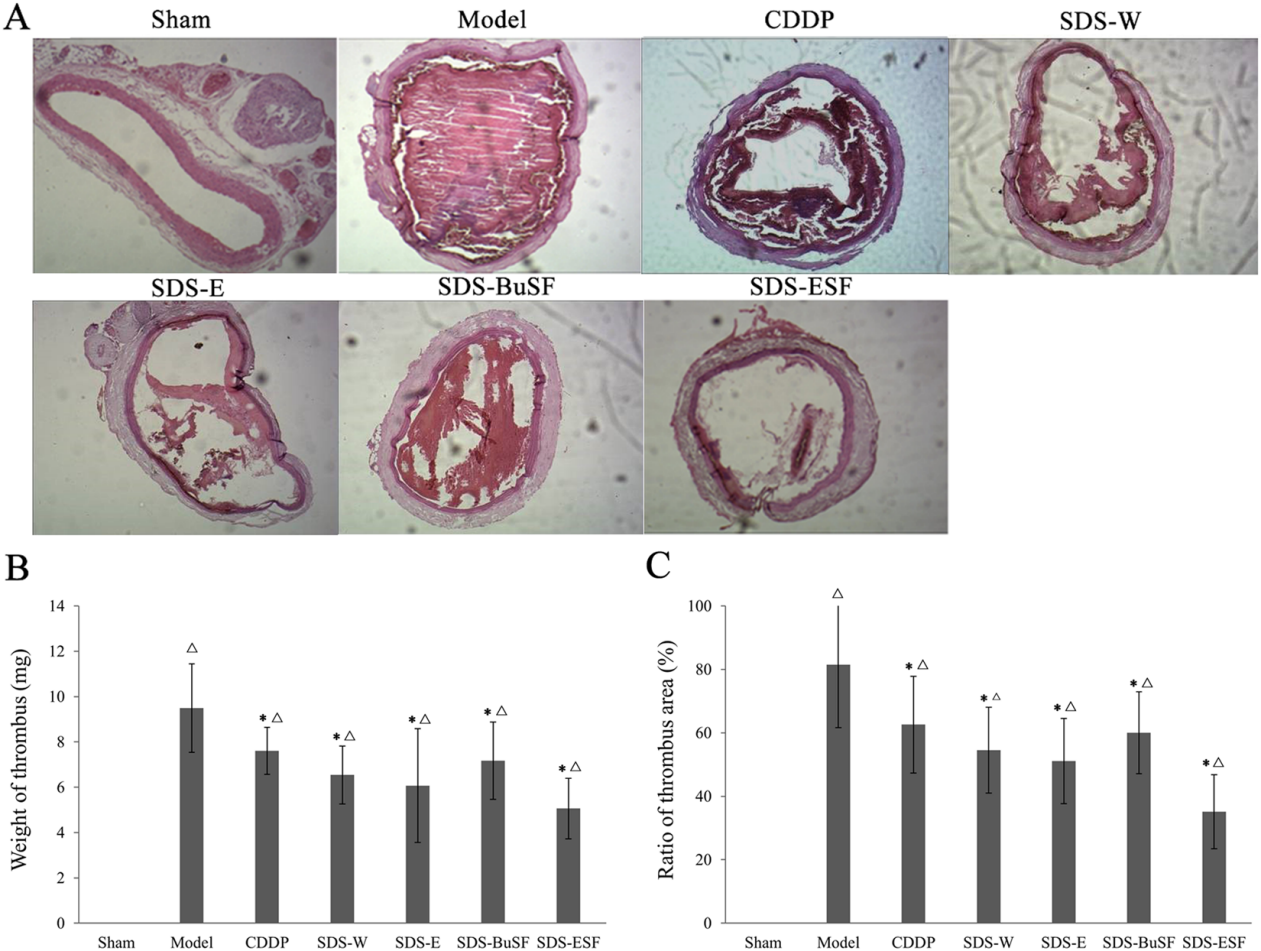
Extracts of SDS roots significantly inhibited FeCl_3_-induced rat carotid artery thrombosis. (**A**) HE staining of rat carotid artery thrombosis in each group; (**B**) comparison of the thrombus weight in each group; (**C**) comparison of the ratio of thrombosis area in each group. Before the establishment of FeCl_3_-induced rat carotid artery thrombosis models, the rats in each group were treated with the indicated solvent extract of SDS roots or CDDP for 20 days, once a day. *n* = 10, data are presented as the mean ± SD; Significant difference compare to model group *P < 0.05, and compre to sham group ^Δ^*P* < 0.05.

Rats in the sham group showed no common carotid endovascular thrombosis, while FeCl_3_ successfully induced rat endovascular thrombosis in the other groups, with weights up to 9 times that of the sham group. (Fig. 2). Compared with the model group, the thrombi in the CDDP and SDS groups were significantly smaller and the thrombus bodies were looser, especially in the SDS root ESF group. Consistent with the morphological results, thrombus weights in each test and CDDP group were significantly lower than that in the model group and thrombosis was the least in the SDS root ESF group (*P* < 0.05, Fig. 2B). In addition, the ratio of thrombus area in each test and CDDP were all significantly lower than that in the model group, expecially SDS root ESF group showed lowest arear ratio (*P < 0.05, Fig. 2C).

### Effects of different SDS root ESF doses on rat carotid artery thrombosis

**Figure 3:**
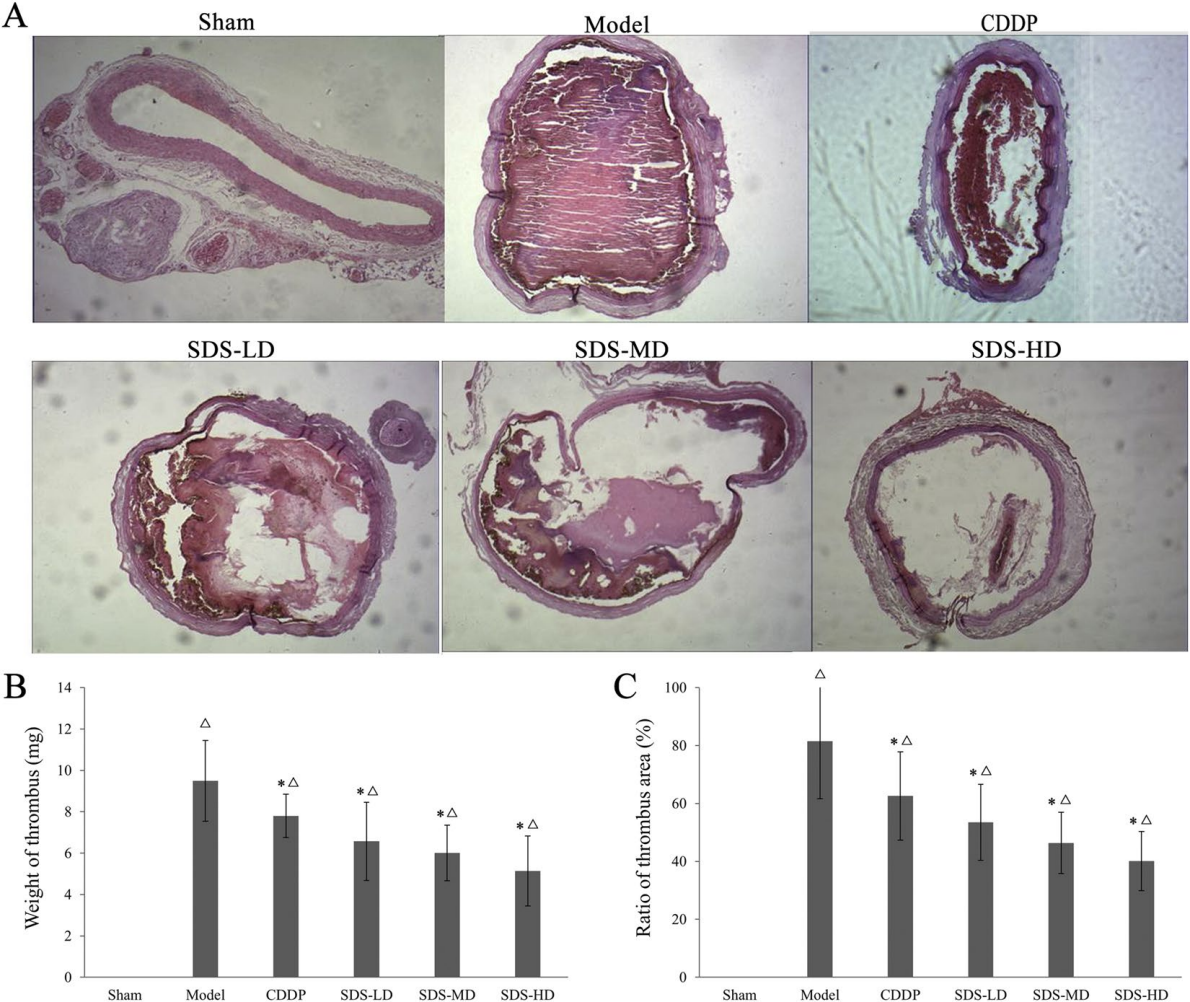
Different doses of SDS root ESF suppressed FeCl_3_-induced rat carotid artery thrombosis. (**A**) HE staining of rat carotid artery thrombosis in each group; (**B**) comparison of the rat thrombus weight in each group; (**C**) comparison of the ratio of thrombosis area in each group. Before the establishment of FeCl_3_-induced rat carotid artery thrombosis models, rats in each group were treated with different doses of SDS root ESF or CDDP for 20 days, once a day, *n* = 10; data are presented as the mean ± SD; *P < 0.05, significant difference compared to the model group; ^Δ^*P* < 0.05 compared to the sham group.

We further compared the inhibitory effects on thrombosis of different doses of SDS root ESF. As shown in Fig. 3A, the low, middle and high dose groups (40 mg/kg, 80 mg/kg and 160 mg/kg, respectively) of SDS root ESFs all significantly inhibited FeCl_3_-induced thrombosis in a dose-dependent manner, losing and/or reducing the weight and area ratio of thrombus bodies (*P* < 0.05, Fig. 3B,C). It is noteworthy that thrombus weight and area ratio in the high dose SDS group was significantly lower than that in the CDDP group, suggesting that its inhibitory effect on thrombosis was stronger than that in the CDDP group (Fig. 3B,C).

### Influences of SDS root ESF on rat plasma ET-1, TXB2, 6-keto-PGF1α and vWF levels

**Figure 4:**
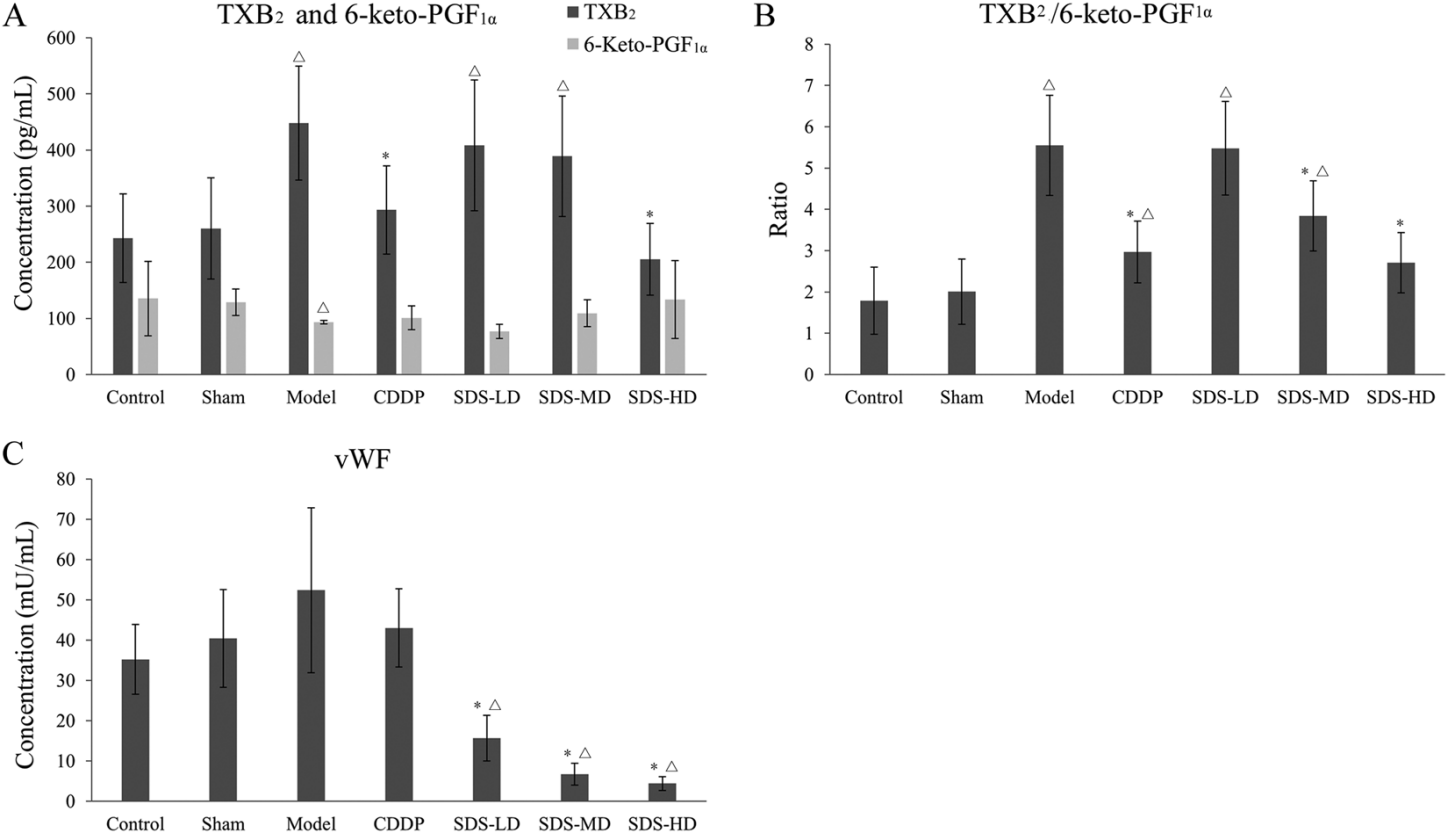
Effects of SDS root ESF on FeCl_3_ induced rat plasma levels of TXB_2_ and 6-keto-PGF_1α_. (**A**) TXB_2_/6-keto-PGF_1α_ ratio; (**B**) and vWF (**C**). Before the establishment of FeCl_3_-induced rat carotid artery thrombosis models, rats in each group were lavaged with different doses of SDS root EtOAc extract or CDDP for 20 days, once a day. *n* = 10; data are presented as the mean ± SD; *P < 0.05, significant difference compared to the model group; ^Δ^*P* < 0.05 compared to the sham group.

TXB_2_ and 6-keto-PGF_1_α are stable metabolites of TXA_2_ and prostaglandin I_2_ (PGI_2_), respectively. TXA_2_ is mainly produced by platelets and is a vasoconstrictor that also stimulates platelet aggregation *in vivo*; vascular endothelial cells mainly produce PGI_2_. The function of PGI_2_ is opposite to that of TXA_2_ with a strong effect on expanding capillaries and inhibiting platelet aggregation. TXA_2_/PGI_2_ imbalance is one cause of platelet aggregation, vascular spasm or thrombosis. As shown in Fig. 4A, compared with the sham group, plasma TXB_2_ levels were significantly higher (*P* < 0.05) and 6-keto-PGF1α levels significantly lower (*P* < 0.05) in the model group, which resulted in a significantly increased TXB_2_/6-keto-PGF_1α_ ratio in the model group (*P* < 0.05, Fig. 4B). CDDP significantly reduced plasma TXB_2_ levels (*P* < 0.05) and the TXB_2_/6-Keto-PGF_1α_ ratio (*P* < 0.05) compared to the model group. Similarly, the high dose group of SDS significantly reduced plasma levels of TXB_2_ (*P* < 0.05) and its effects were somewhat stronger than CDDP.

von Willebrand factor (vWF) is a multimeric plasma protein that mediates platelet adhesion as well as platelet aggregation at sites of vascular injury and acts as a carrier of factor VIII^18^. Compared with the sham group, the vWF level in the model group was significantly increased (*P* < 0.05). CDDP treatment appeared to reduce the increase in vWF concentration compared to the model group, but statistical significance was not achieved, whereas SDS treatment significantly suppressed the increase of vWF concentrations (*P* < 0.05) in a dose-dependent manner (Fig. 4C).

### Influence of SDS root ESF on rat plasma PC and AT-III levels

**Figure 5:**
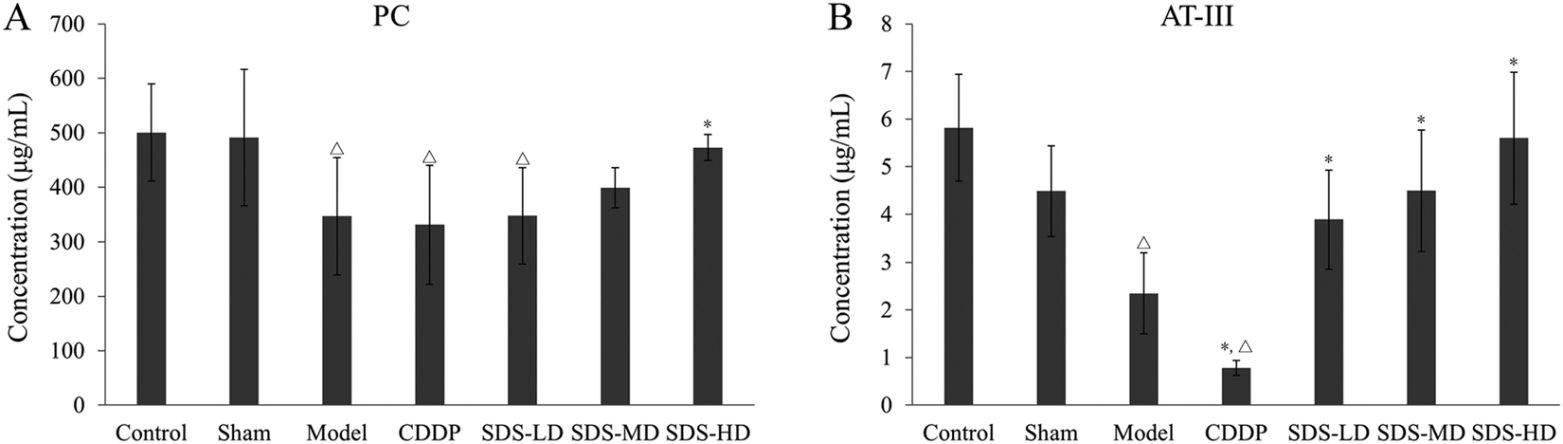
Effects of SDS root EtOAc extracts on FeCl_3_-induced rat plasma levels of PC. (**A**) and AT-III (**B**). Before the establishment of the FeCl_3_-induced rat carotid artery thrombosis models, rats in each group were treated with different doses of SDS root EtOAc extract or CDDP for 20 days, once a day, *n* = 10; data are presented as the mean ± SD; *P < 0.05, significant difference compared to the model group; ^Δ^*P* < 0.05 compared to the sham group.

Protein C (PC) system and AT-III are important physiological anticoagulants *in vivo*. Compared with the sham group, PC (*P* < 0.05, Fig. 5A) and AT-III (*P* < 0.05, Fig. 5B) were significantly reduced in the model group. Compared with the model group, the AT-III concentration decreased significantly in the CDDP group (*P* < 0.05), but the PC concentration did not change. In contrast, compared to the model group the PC (*P* < 0.05, Fig. 5A) and AT-III (*P* < 0.05, Fig. 5B) serum concentrations were significantly increase particularly in the high dose group.

### Influences of SDS root ESF on rat plasma PLG, t-PA and PAI-1

**Figure 6:**
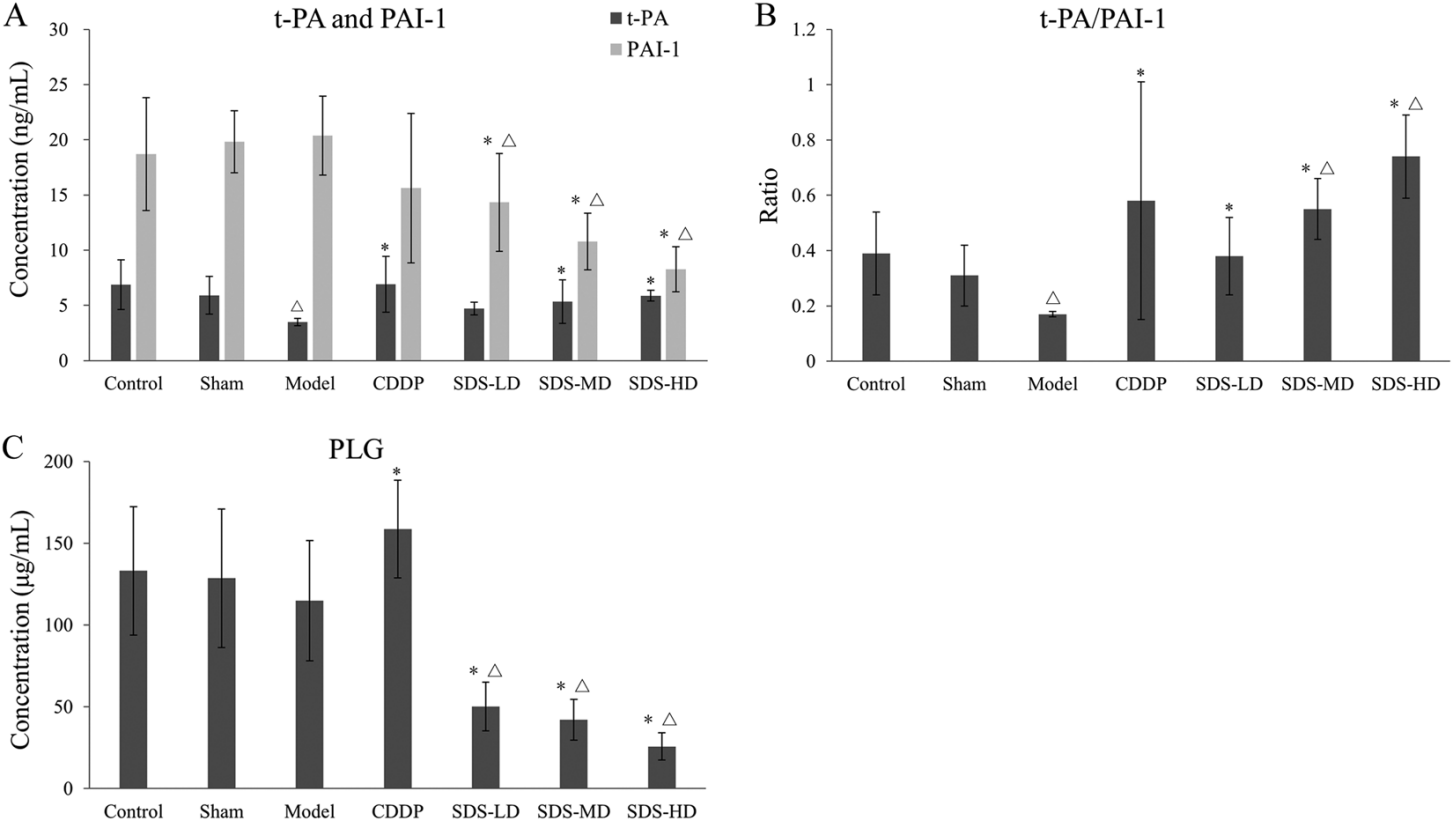
Effect of SDS root ESF on the activity of the fibrinolytic system. Influences of each dose of EtOAc extract on FeCl_3_-induced rat plasma levels of (**A**) t-PA and PAI-1, (**B**) t-PA/PAI-1 ratios and (**C**) PLG. Before the establishment of FeCl_3_-induced rat carotid artery thrombosis models, rats in each group were treated with different doses of SDS root EtOAc extract or CDDP for 20 days, once a day, *n* = 10; data are presented as the mean ± SD; *P < 0.05, significant difference compared to the model group; ^Δ^*P* < 0.05 compared to the sham group.

Plasminogen (PLG) is the precursor of plasmin, which is a fibrin hydrolase and mainly produced by the actions of the serum tissue-type plasminogen activator (t-PA). The resulting plasmin dissolves fibrin in blood clots. In contrast, the plasminogen activator inhibitor (PAI-1) can inhibit the activation of t-PA. Compared with the sham group, the plasma t-PA/PAI-1 ratio showed significant decrease (*P* < 0.05, Fig. 6B), while the PLG concentration did not show a significant decrease in the model group (Fig. 6C) and the t-PA concentration was significantly reduced (*P* < 0.05, Fig. 6A). Compared with the model group, PLG (*P* < 0.05) and t-PA (*P* < 0.05) concentrations in the CDDP group were increased and the PAI-1 concentration was decreased, resulting in a significantly increased t-PA/PAI-1 ratio (*P* < 0.05, Fig. 6B). Although in contrast to the CDDP group, PLG concentrations showed no enhancement but rather significant decreases (*P* < 0.05), the PAI-1 concentrations in each SDS dose group were significantly decreased and the concentration of t-PA significantly increased in a dose-dependent manner, resulting in significantly increased t-PA/ PAI-1 ratios in all dose groups (Fig. 6C).

### Horminone, 7-O-acetylhormione and 6,7-dehydeoroyleanone were active ingredients in SDS root extracts

In order to further analyze active ingredients in SDS, we subjected SDS extracts to a HPL-C/CG-MS analysis and found that horminone, 7-O-acetylhormione and 6,7-dehydroroyleanone were compounds (Supplementary Figure 2). As shown in Table 2, apart from low doses of 6,7-dehydroroyleanone and 7-O-acetylhorminone all other doses of the 3 tested chemicals led to significant reductions in MAIR% compared to the controls.

**Table 2:** Effects of 6,7-dehydroroyleanone, horminone and 7-O-acetylhorminone on platelet aggregation (*n* = 7, $\overline{x}$ ± *s*).

	Group	Concentration/µg/mL	MAR/%	MAIR/%
6,7-dehydroroyleanone	Aspirin	100	16.7 ± 4.31	62.3 ± 11.5
	Control	—	97.1 ± 1.86^Δ^	—
	High	1000	50.6 ± 10.20*^Δ^	47.8 ± 10.5^Δ^
	Medium	100	73.0 ± 5.90*^Δ^	24.7 ± 6.07^Δ^
	Low	10	94.4 ± 6.08^Δ^	1.20 ± 5.31^Δ^
	*p-*value (Dose)		<0.0001	
	*p-*value (Aspirin + Dose)		<0.0001	<0.0001
Horminone	Aspirin	100	17.35 ± 4.63	61.70 ± 5.82
	Control	—	91.00 ± 2.89^Δ^	—
	High	1000	66.10 ± 4.80*^Δ^	27.50 ± 5.28^Δ^
	Medium	100	79.10 ± 1.21*^Δ^	13.00 ± 1.36^Δ^
	Low	10	85.80 ± 1.68*^Δ^	6.10 ± 2.13^Δ^
	*p-*value (Dose)		<0.0001	
	*p-*value (Aspirin + Dose)		<0.0001	<0.0001
7-O-acetylhorminone	Aspirin	100	18.03 ± 5.06	58.63 ± 8.91
	Control	—	65.1 ± 7.15^Δ^	—
	High	1000	42.60 ± 7.02*^Δ^	34.50 ± 10.80^Δ^
	Medium	100	51.80 ± 6.25*^Δ^	17.80 ± 4.70^Δ^
	Low	10	63.40 ± 4.93^Δ^	2.40 ± 7.60^Δ^
	*p-*value (Dose)		<0.0001	
	*p-*value (Aspirin + Dose)		<0.0001	<0.0001

*P < 0.05 significant difference compared to control group; ^Δ^*P* < 0.05 significant difference compared to aspirin group.

MAIR (%) = platelet aggregation rate of the control group% - platelet aggregation rate of test drug treated group% / platelet aggregation rate of the control group%.

## Discussion

In the present study, we extracted different plant parts of SDS using a number of polar solvents, including water, ethanol n-Butanol and EtOAc, and then determined the antithrombotic effects of SDS extracts in New Zealand white rabbit anti-platelet aggregation and FeCl_3_-induced rat carotid artery thrombosis models.

The anti-platelet aggregation effective substances were rich in root SDS and EtOAc was the effective extraction solvent for it. In the New Zealand white rabbit anti-platelet aggregation model, SDS root ESF (40 mg/kg) significantly inhibited ADP-induced platelet aggregation, which was equivalent to the anti-platelet aggregation effects of aspirin (10 mg/kg). These findings suggested that SDS root extracts have similar anti-platelet aggregation effects as *Salvia miltiorrhiza bunge*^19^. Also in the FeCl_3_-induced rat common carotid artery thrombosis model group, particularly high doses of SDS root extracts inhibited thrombus development, which is in agreement with a previous study in which anti-platelet drugs could extend the time until occlusion in a FeCl_3_ common carotid artery thrombosis model^20^. It is noteworthy that this effect was more pronounced with SDS root ESF than with CDDP (Figs 2 and 3). Consistent with a previous study, we observed that the serum concentration of TXB_2_ increased in the FeCl_3_ common carotid artery thrombosis model group, while the activities of 6-keto-PGF_1_α, t-PA, AT-III and PC were reduced^21^. Moreover, treatment particularly with high doses SDS root ESF reduced plasma TXB_2_ and vWF, and increased 6-keto-PGF_1_α, leading to a significantly reduced TXB_2_/6-keto-PGF_1α_ ratio, which was similar to the effect of CDDP and underlined the anti-platelet aggregation effects of SDS extract shown in the rabbit and rat models. However, in contrast to the CDDP formulation, vWF expression was significantly and dose-dependently reduced in SDS treated rats, indicating that SDS might have a stronger antithrombotic effect than CDDP (Fig. 4). As an indicator of coagulation inactivation, PC and AT-III serum levels were increased in SDS treated rats with the highest levels found in the SDS-HD group, indicationg a trigger of anti-coagulation by SDS root extracts.

These data are contrary to the CDDP-induced changes, since AT-III serum concentrations were significant lower and PC serum levels remained the same as the model group in CDDP treated rats (Fig. 5). Taken together, the antithrombotic effect of SDS root ESF can be attributed to anti-platelet activity and anti-coagulation actions, similar to but not the same as CDDP. However, these findings supported our hypothesis that SDS can induce similar effects on thrombus development as *Salvia miltiorrhiza bunge*.

In order to confirm the platelet aggregation inhibitory effect of 6,7-dehydroroyleanone^11^ and the two other diperpenoid quinones, horminone and 7-O-acetylhormione, which we isolated from SDS roots and which have been described as constituents in a previous study^9^, we carried out a dose increasing experiment in rabbits and found that all 3 chemicals significantly inhibited platelet aggregation in a dose-dependent manner (Table 2). Tanshinone IIA is a diperpenoid quinone in *Salvia miltiorrhiza bunge* and has been attributed to be a major active compound of Danshen with antiplatelet and anticoagulant effects via tubulin acetylation and Erk-2 phosphorylation inhibition^22^. However, whether the same mechanisms are valid for 6,7-dehydroroyleanone, horminone and 7-O-acetylhormione actions requires further investigation, while other compounds may also have an effect on blood coagulation.

One drawback of our study was that the time between injury and data collection was short, which may have had an influence, particularly on fibrinolytic enzyme data.

## Conclusion

In conclusion, the present study demonstrated that SDS root ESF had significant antithrombotic effects, with EtOAc being the most effective extraction solvent. The antithrombotic effect could be attributed to enhanced anti-platelet aggregation and coagulation inactivation. Thus, further studies on SDS ESF as an antithrombotic traditional Chinese medicine are warranted.

## Electronic supplementary material

Supplementary materials

## Data Availability

The datasets supporting the conclusions of this article is included within the article.
