# Supplementary material for: Antithrombotic effects and related mechanisms of Salvia deserta Schang root EtOAc extracts
Source: Sci Rep. 2018 Dec 10;8:17753. doi: 10.1038/s41598-018-36026-7 (PMC6288089; doi:10.1038/s41598-018-36026-7)
Supplement: Supplementary file 1 — Supplementary materials [file 41598_2018_36026_MOESM1_ESM.docx]

**Antithrombotic effects and related mechanisms of *Salvia deserta Schang* root EtOAc extracts**

**Running title:** Antithrombotic effects of SDS root extracts

Rena Kasimu^1,3^, Xinling Wang^1*^, Xiaomei Wang^1^, Junping Hu^1^, Xiaoqing Wang^1^, Yuming Mu^2^

^1^College of Pharmacy, Xinjiang Medical University. No.393 Xinyi Road, Urumqi, 830011, China.

^2^The First Affiliated Hospital Of Xinjiang Medical University, No.137 South Liyushan Road, Urumqi, 830054, China

^3^Deceased.

***Correspondence:**

**Xinling Wang**

College of Pharmacy, Xinjiang Medical University. No.393 Xinyi Road, Urumqi, 830011, China

Tel: +86-991-4362473

Fax: +86-991-4362473

E-mail: [365021216@qq.com](mailto:365021216@qq.com)

**Supplementary materials**

**Supplementary Table 1**: Doppler ultrasound data (ml/min) of common artery blood flow in rats after exposure to high (2.16 mol/L) and low (1.08 mol/L) FeCl_3_ concentration solutions.

| Time of FeCl_3_ treatment | High FeCl_3_ concentration | Low FeCl_3_ concentration |
| --- | --- | --- |
| Before treatment | 20.98±4.89 | 20.46±4.76 |
| 5 min | 9.21±5.24 | 16.27±4.54 |
| 10 min | 6.26±5.63 | 13.08±4.39 |
| 15 min | 0.28±0.23 | 12.07±4.55 |
| 20 min | 0.07±0.09 | 11.75.±7.56 |
| 30 min | 0.06±0.01 | 11.43±7.45 |
| 40 min | 0.07±0.03 | 11.23±7.14 |
| 60 min | 0.05±0.04 | 11.36±7.56 |


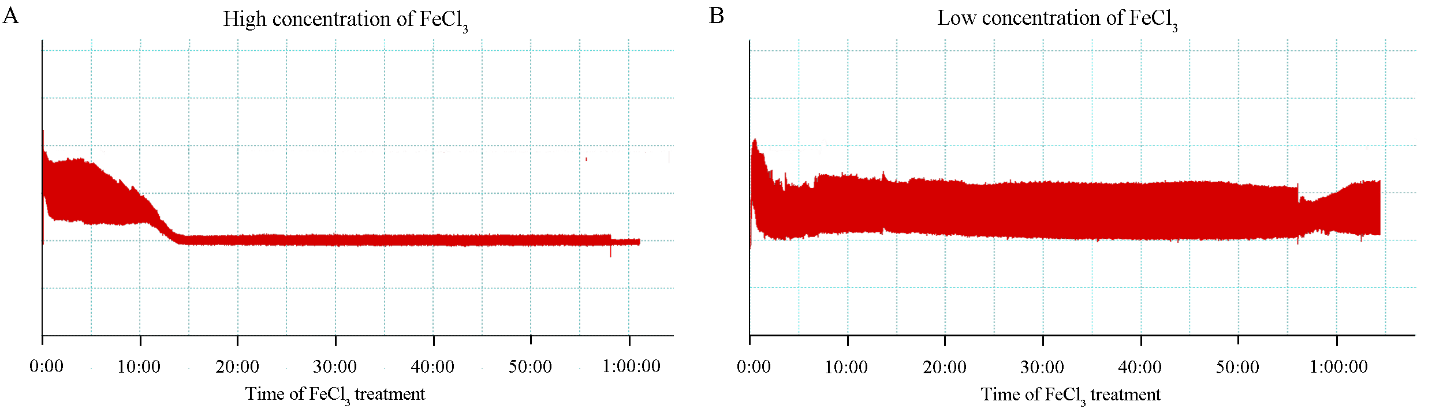


**Supplementary figure 1:** Doppler ultrasound graph of common carotid artery blood flow after exposure to A) high (2.16 mol/L) and B) low (1.08 mol/L) doses of FeCL_3_


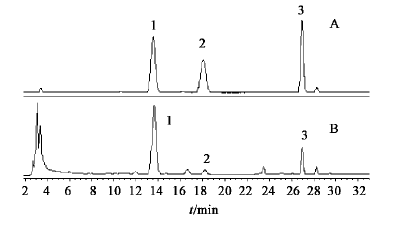


**Supplementary figure 2:** HPLC (high performance liquid chromatography) analysis of Salvia deserta roots. A) control sample; B) SDS sample; 1: horminone, 2:7-Oacetylhorminone, 3:6, 7-dehydroroyleanone.
